# Open Lumbar Spine Image Analysis: A 3D Slicer Extension for Segmentation, Grading, and Intervertebral Disc Height Index With Multi–Data Set Validation

**DOI:** 10.1097/BRS.0000000000005462

**Published:** 2025-08-01

**Authors:** Narasimharao Kowlagi, Antti Kemppainen, Terence McSweeney, Simo Saarakkala, Jérôme Noailly, Frances M.K. Williams, Jason Pui Yin Cheung, Jaro Karppinen, Aleksei Tiulpin

**Affiliations:** a Research Unit of Health Sciences and Technology, University of Oulu, Oulu, Finland; b Department of Diagnostic Radiology, University of Oulu Hospital, Oulu, Finland; c Department of Information and Communication Technologies, Universitat Pompeu Fabra, Barcelona, Spain; d Department of Twin Research and Genetic Epidemiology, King’s College London, London, UK; e Department of Orthopaedics and Traumatology, The University of Hong Kong, Hong Kong SAR, China; f Rehabilitation Services of Wellbeing Services County of South Karelia, Lappeenranta, Finland

**Keywords:** disc height index, 3D Slicer, external validation, lumbar spine grading, lumbar spine segmentation

## Abstract

**Study Design.:**

Retrospective and cross-sectional study.

**Objective.:**

The study aims to develop an open software for lumbar spine image analysis enabling no-code approach to lumbar spine segmentation, grading, and intervertebral Disc Height Index (DHI) calculations with robust evaluation of the application on 6 external data sets from diverse geographical regions.

**Background.:**

The data sets used include NFBC1966 (Finland), HKDDC (Hong Kong), TwinsUK (UK), CETIR (Spain), NCSD (Hungary), SPIDER (Netherlands), and Mendeley (global). Thirty participants from each data set were sampled for external evaluation, and NFBC1966 was used for training. Annotation was performed on T2-weighted mid-sagittal slices of vertebral bodies L1 to S1 and intervertebral discs L1/2 to L5/S1.

**Materials and Methods.:**

Open Lumbar Spine Image Analysis (OLSIA) application was developed to include no-code approach tools for automated segmentation, grading, DHI calculation, and batch processing capabilities by integrating the deep learning (DL) models. DL models were trained on the NFBC1966 data set with augmentation (histogram clipping, median filtering, and geometric scaling) to improve generalization. Interrater agreement was assessed using dice similarity coefficient (DSC), Bland-Altman (BA) analysis for DHI measurements and a paired *t* test for statistical significance.

**Results.:**

Our application demonstrated 222-fold improvement in processing time compared with performing manually lumbar spine segmentation, grading and DHI calculation tasks. OLSIA’s segmentation performance exhibited close correspondence with the interrater agreement across all 6 external data sets. Interrater reliability was high (mean DSC >90). Although paired *t* test on DHI measurements is significant (*P* < 0.05), the mean difference (0.02) of DHI from the BA plots indicates low systematic bias.

**Conclusion.:**

We introduced OLSIA, a user-friendly interface for lumbar spine segmentation, grading, and intervertebral DHI calculation. OLSIA empowers researchers from diverse backgrounds to efficiently use the no-code tools to accelerate their radiomics and lumbar spine image analysis workflows.

Medical imaging is vital in the detection of anomalies, diagnosis of disease, and in phenotyping.^1^ As such, imaging of the lumbar spine is used to determine the need for surgical intervention and to investigate various aspects of spine pathology, including the etiology of low back pain.^2^ Traditional manual and qualitative reporting of imaging findings is currently being superseded by radiomics and deep learning (DL)-driven image analysis.^3^ In spine image analysis, the use of DL-based models has shown promising results in several applications including spine segmentation,^4–8^ grading,^4,8–11^ reconstruction,^12,13^ and surgical planning.^14,15^

Radiomics, a quantitative approach to image analysis, can be applied to different medical image modalities, such as plain radiographs, computed tomography (CT), and magnetic resonance imaging (MRI).^16^ Through this method, high-dimensional features are extracted from medical images,^3^ which together with clinical and omics data, can be used to develop complex models.^17^ These models can aid in deciphering complex disease relationships and improve diagnostic accuracy.^3^ Radiomics has also been applied to the spine, osteoporosis detection,^18^ vertebral compression fracture prediction,^19^ spine lesion classification,^20^ and spine surgery.^21^

Generally, the first step in a radiomics pipeline is to identify regions of interest (ROIs) in the image for which high-dimensional features are extracted.^16^ This is achieved using automated image segmentation tools^18,22^ or manual annotations.^23–25^ However, the latter is time-consuming and often limited to small data sets.^8^ Recent studies have explored the use of DL models for automatic segmentation of spine imaging within the radiomics pipeline.^22,26,27^ This approach involves training DL models specifically for segmentation tasks such as vertebral body detection.^26,27^ Several DL models have been developed for spine segmentation tasks.^8,9,28,29^ However, the use of these models poses several challenges. First, when these models are not publicly available, implementing the proposed DL architecture requires considerable technical expertise, and can prove challenging.^30^ Second, even when the models are published for research use, exploiting their full potential often necessitates intermediate expertise,^30^ including scripting^22^ and complex installation.^31^

To mitigate these limitations, we developed the Open Lumbar Spine Image Analysis (OLSIA) platform for streamlined lumbar spine MRI (LSMRI) analysis. OLSIA integrates DL models^9^ for lumbar spine segmentation and Pfirrmann grading (Figure 1) and performs robust, automatic calculation of disc height index (DHI), a commonly used measure of disc health^32^ in research. We validated OLSIA’s robustness using 6 diverse external data sets. OLSIA aims to improve the accessibility of LSMRI analysis and accelerate low back pain research.

**Figure 1:**
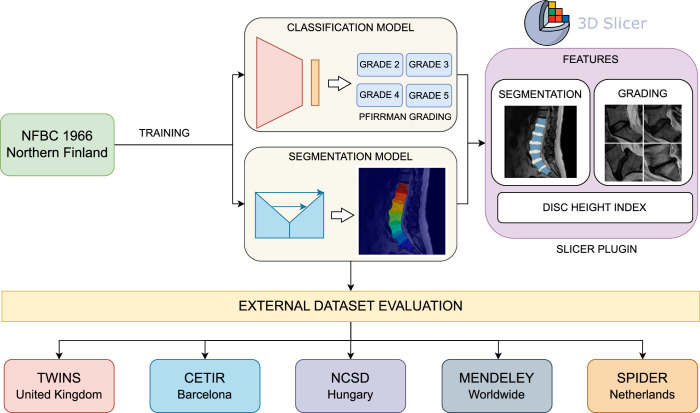
Overall pipeline demonstrating the training and validation of the deep learning models on different data sets and no-code features that are packaged into Open Lumbar Spine Image Analysis application.

## MATERIALS AND METHODS

### OLSIA Extension

To encourage wide adoption, we developed OLSIA as an extension to 3D Slicer (Version 5.6.2),^33^ a popular open-source biomedical image analysis software. OLSIA has 3 main features: First, we integrated the DL models for segmenting lumbar vertebral bodies (L1–S1) and intervertebral discs (L1/2–L5/S1). Second, we incorporated a report that includes both the lumbar spine grading for each disc using Pfirrmann grading,^34^ and the DHI. Finally, batch processing of DICOM images is built into the application by integrating the 3D Slicer’s DICOM database enabling automatic generation of segmentation masks and reports (Pfirrmann grade, DHI) for multiple participants.

### Cross-Geographic Data Set Evaluation

#### Data Sets

This study utilized 7 distinct lumbar spine data sets (Table 1) from diverse geographical locations. The Northern Finland Birth Cohort 1966^35,36^ (NFBC1966), consisting of MRI scans obtained between 2012 and 2015 from individuals born in Northern Finland in 1966, served as the primary data set for model training. To assess the generalizability of the trained model rigorously, 6 additional data sets were incorporated: the Hong Kong Disc Degeneration Cohort (HKDDC)^37^ from Hong Kong, TwinsUK^38^ from the United Kingdom, CETIR^39^ from Spain, NCSD^39^ from Hungary, and the SPIDER data set from the Netherlands^5^ and a collection of scans from around the world published on Mendeley.^40^ The private MRI data sets were collected by the partnering institutions with informed consent from the participants. Comprehensive details on the scan parameters and the manufacturer information for each data set are provided in the supplementary information (Supplemental Digital Content 1, http://links.lww.com/BRS/C790).

**TABLE 1:** Number of Samples and the Country of Origin for Each Data Set

Data Set	No. Samples	Country
NFBC1966	1500	Finland
TwinsUK	996	United Kingdom
HKDDC	632	Hong Kong
CETIR	30	Spain
NCSD	30	Hungary
Mendeley*	515	Around the world
SPIDER*	257	Netherlands

* Data are publicly available.

#### Data Selection and Annotations

The data sets were preprocessed to ensure a robust validation setup across all 7 data sets with varying sample sizes. To establish a consistent cohort size, a random sample of 30 participants was selected from the larger data sets (NFBC1966, TwinsUK, HKDDC, Mendeley, and SPIDER). In contrast, all 30 participants from the smaller CETIR and NCSD data sets were included in the analysis. This strategy facilitated direct comparative analysis by ensuring uniform sample sizes across all the data sets, mitigating potential biases introduced by the sample size variability.

Vertebral bodies (L1–S1) and intervertebral discs (L1/2–L5/S1) were annotated on mid-sagittal T2-weighted, fat-suppressed MR images. We created segmentation masks for these ROIs using ITK-SNAP (Version 4.0.1, University of Pennsylvania, Philadelphia, Pennsylvania, USA) annotation software. Although certain data sets included the entire DICOM series, annotations were performed exclusively on the T2-weighted mid-sagittal slice. This approach was adopted for 2 primary reasons: first, our segmentation and grading model was specifically trained for the mid-sagittal region. Second, this methodology ensured consistency with data sets limited to mid-sagittal images, thereby promoting uniformity in the anatomic regions analyzed across all data sets.

#### Intervertebral Disc Height Index

DHI is a quantitative measure that can be computed from segmentation masks of LSMRI.^32^ Studies have indicated a correlation between the DHI and the incidence of recurrent lumbar intervertebral disc herniation^41^ and facet joint osteoarthritis^42^ making it an important measurement. Using the segmentation masks, we calculated DHI for levels L1/2 to L5/S1 with the formula:


$$\mathrm{Disc Height Index}=\frac{2\;*\mathrm{IVD Height}}{\left(\mathrm{Cranial VB Height}+\mathrm{Caudal VB Height}\right)}$$

Where IVD refers to the intervertebral disc and VB refers to the vertebral body of the lumbar spine. Computing DHI is typically time-consuming and requires measurements of VB and IVD heights using DICOM viewer, followed by manual calculation. Therefore, the inclusion of this feature in the application facilitates accelerated DHI extraction and demonstrates a technical workflow for bringing other features.

#### Interrater Agreement

To estimate DHI measurement variability, we conducted an interrater agreement study using manual segmentations from 2 annotators: A1 (board-certified radiologist, 5 yr of experience) and A2 (MSK researcher, MRI annotation experience). Agreement was evaluated using 3 metrics. First, dice similarity coefficient (DSC) compared A1/A2 annotations across 5 external data sets (excluding HKDDC due to A1 access limitations). Second, Bland-Altman analysis^43^ assessed agreement and bias in DHI derived from segmentations; statistical significance was checked using a paired *t* test. Finally, variance was measured using R^2^ with 95% CIs from 1000 bootstrap resamples.

### OLSIA Performance Evaluation

We evaluated OLSIA’s efficiency by comparing its processing time against manual segmentation, grading, and DHI calculation. Raters A1 and A2 were presented with 3 patients, and the time taken to annotate each participant was recorded. To determine the average time for manual segmentation, we calculated the arithmetic mean of these recorded times.

As per grading and DHI, 2 musculoskeletal (MSK) researchers (J.K. with over 30 yr of experience and T.M. with 10 yr of experience as an osteopath) provided their individual estimates for manual grading and DHI calculations. The arithmetic mean of their individual estimates was then chosen as the average time required for these tasks. In addition, we evaluated the segmentation masks predicted by OLSIA against the annotations made by A1 and A2, by using the DSC.

We benchmarked OLSIA’s segmentation performance against TotalSegmentator,^29^ focusing on lumbar vertebral bodies (L1–S1) and intervertebral discs (L1/L2–L5/S1). Segmentation quality was quantified using the Intersection over Union (IoU) metric, which measures the overlap between the segmentations produced by OLSIA and the reference segmentations from TotalSegmentator^29^ for each target structure.

For robust performance evaluation, we tested OLSIA on 3 diverse systems: (M1 - Windows 10 22H2, i7-10510U with 16GB RAM; M2 - Ubuntu 24.04, AMD Ryzen 9 5950X, 128GB RAM, NVIDIA RTX A4000 32GB GPU; M3 - Mac Mini, Mac OS Monterey 12.6.2, Intel Core i5, 8GB RAM). On each computer, we recorded times for individual segmentation, grading, and DHI calculation, along with the time for batch processing 30 participants.

### Lumbar Spine Models Generalization

Our DL component builds upon our previous work on optimal model architectures.^9^ We retrained these models on the NFBC1966 data set, applying 3 additional augmentations to improve generalization across the 6 external data sets: The first augmentation consisted of applying histogram clipping, to limit pixel values to the 0.5th and 99.5th percentiles and eliminate extremely bright and extremely dark regions that lack detail. Second augmentation aimed to control variability in image intensity by applying a median filter with a kernel size of 11, which introduces slight random variations in intensity values. This process enhanced the model’s robustness to variations in lighting, contrast, and imaging conditions. Finally, we used geometric scaling to present the model with images of different spatial dimensions, a critical augmentation given the variability in MRI image geometry across imaging centers. Details of model architecture are provided in the supplementary (Supplemental Digital Content 1, http://links.lww.com/BRS/C790).

To further enhance generalization, we used weighted batch normalization (WBN).^44^ This technique effectively balances the influence of each data set, ensuring that the model statistics are learned from the overall patient population rather than from the characteristics of a single data set. The models were trained on the NFBC data set using a 5-fold cross-validation approach over 150 epochs, and the training was stopped when no improvement in performance was achieved for 20 consecutive epochs. Pytorch^45^ (Version: 1.12.1) framework was utilized for model development. All the experiments and model training were conducted on 2 x 16GB NVIDIA RTX A4000 Graphical Processing Units (GPUs).

## RESULTS

### OLSIA Platform Evaluation

Among the 3 manual tasks, DHI calculation was the most time-consuming, requiring manual measurement of intervertebral disc and vertebral body heights using a DICOM viewer and subsequent calculation of the DHI. This process required an average of 15 minutes per participant, notably longer than manual annotation and grading, which required on average 3 minutes.

In contrast, when using OLSIA on the M2 configuration for a single participant, the segmentation task completed in 2.29 ± 0.01 seconds, while the grading and DHI calculations together took 3.37 ± 0.02 seconds. This represents a 222-fold reduction in processing time compared with the manual approach. Even with the M1 and M3 configurations, which have only CPUs and lower memory, OLSIA still demonstrated a considerable advantage (13 s on average per participant), achieving a 97-fold increase in speed compared with manual processing. In batch processing mode, analysis of the data of the 30 participants was completed in <7 minutes using the M1 and M3 configurations, and in <3 minutes using the higher-performance M2 configuration (Table 2).

**TABLE 2:** Comparison of Timings for Segmentation, Grading and Disc Height Index (DHI) Calculation Tasks Using OLSIA *Versus* Manual Approach

			OLSIA Batch Processing		
Feature	Manual	OLSIA	Windows 10 22H2	Linux Ubuntu 24.04	Mac Monterey 12.6.2
Segmentation	~3	0.03±0.00	6.25±0.03	2.43±0.00	6.58±0.00
Pfirrmann grading	~3	0.05±0.00			
DHI calculation	~15				

All the values indicate the average number of minutes elapsed with the SE between 3 different timings recorded using same subject. For batch processing, a total of 30 participants are used.

To further assess the performance of OLSIA, we evaluated the application over 5 external data sets. First, we computed the DSC between the predictions by OLSIA and the annotations provided by the experienced rater A1. To establish a benchmark for comparison, we also calculated the interrater agreement (DSC) between raters A1 and A2. The results showed close correspondence between the OLSIA’s performance and the interrater agreement, indicating a high level of consistency (Table 3).

**TABLE 3:** Comparison of Segmentation Performance (Dice Similarity Coefficient; DSC) Between the Base Model, the Augmented Model, and the Adapted Model on 6 External Data Sets

Data Set	InterRater Agreement	Base Model	Augmented Model	Adapted Model
CETIR	95.0±0.1	82.6±0.0	95.2±0.0	95.2±0.3
TwinsUK	93.8±0.1	9.5±0.0	86.7±0.0	88.9±0.1
NCSD	94.3±0.1	87.6±0.0	94.0±0.0	94.0±0.0
Mendeley	95.4±0.0	91.2±0.0	92.7±0.0	92.5±0.0
SPIDER	92.9±0.5	86.6±0.0	91.0±0.0	91.6±0.1
HKDDC	—	82.5±1.8	88.7±0.7	90.5±0.2
NFBC1966	—	99.0±0.0	99.2±0.0	99.2±0.0

The base model was trained on the NFBC1966 data set. Interrater agreement (DSC) is also reported for 5 of the data sets.

### Generalization Across Populations

**Figure 2:**
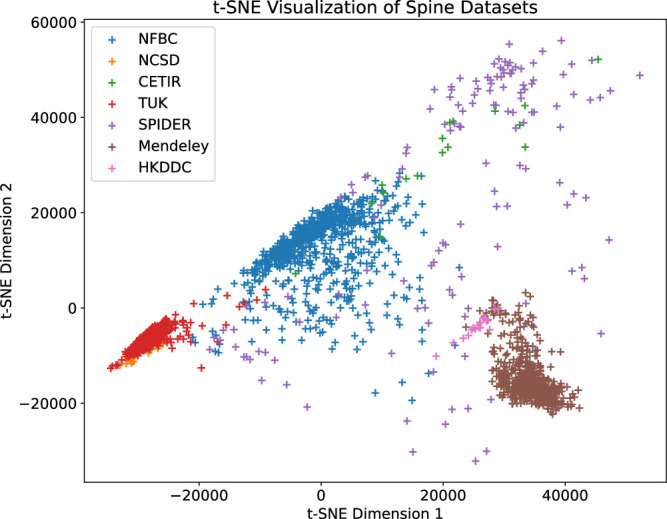
t-distributed Stochastic Neighbor Embedding plot representing the lower-dimensional projection of lumbar spine images from 6 external data sets. Each data set is visualized using a distinct color with “+” marker, demonstrating the relationships and variations between data sets. Clustering patterns may indicate similarity in image features across data sets, while separations reflect potential domain shifts or differences in imaging protocols.

**Figure 3:**
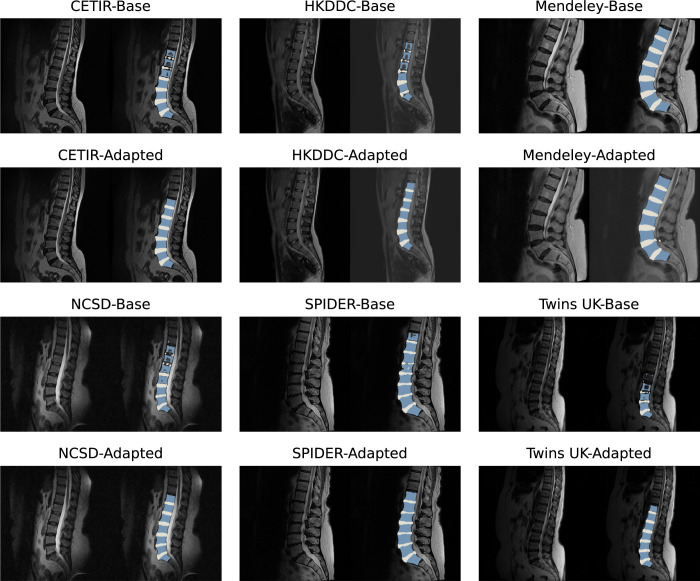
Comparison of segmentation performance between the base and adapted models across 6 external lumbar spine magnetic resonance imaging data sets. The adapted model demonstrates improved generalization providing more precise detection of vertebral bodies and intervertebral discs.

**Figure 4:**
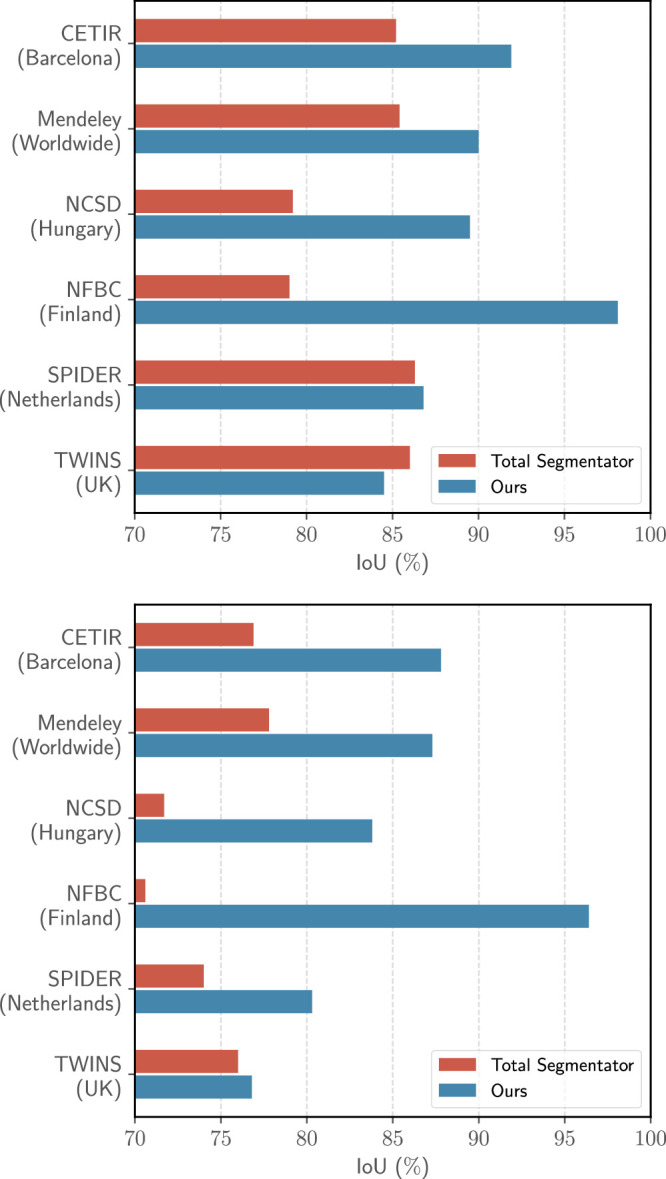
Model comparison between our model and the TotalSegmentator model for vertebral body and disc segmentation. Our model demonstrates superior performance across all data sets, with comparable performance on the TwinsUK and SPIDER data sets, highlighting its robustness and generalizability.

Before evaluating the model performance, we first assessed data set diversity using t-distributed Stochastic Neighbor Embedding (t-SNE) visualization of image embeddings (Figure 2). This dimensionality reduction technique illustrates the relationships between data points in a low-dimensional space, revealing the underlying variability. Subsequently, to evaluate model performance, the final prediction was generated by averaging the outputs of the 5 models produced during 5-fold cross-validation. Compared with the base model,^9^ there was a noticeable improvement in DSC across all data sets (Table 3). We found α = 0.6 as an optimal setting for WBN, which provided the best balance in performance between the training data (NFBC1966) and the 6 external evaluation data sets. A visual comparison of the predictions generated by the base model and the adapted model is presented in Figure 3. In addition, we evaluated the segmentation results of OLSIA against the segmentations obtained using TotalSegmentator.^29^ OLSIA demonstrated superior segmentation performance for vertebral bodies (DSC >5%) and discs (DSC >8%) on most data sets, with comparable performance observed for the TwinsUK and SPIDER data sets (Figure 4).

### Variability in Disc Height Measurements

Interrater reliability analysis of the lumbar spine annotations revealed a high degree of concordance between raters A1 and A2. A mean DSC exceeding 90.0 was observed across all data sets, with slightly lower scores for the TwinsUK and SPIDER data sets. Furthermore, the model’s predictive performance closely approximated the interrater agreement, exhibiting a maximum deviation of 4% below the interrater agreement for the TwinsUK and SPIDER data sets, and an average of 1.3% lower than the interrater agreement for the remaining data sets.

**Figure 5:**
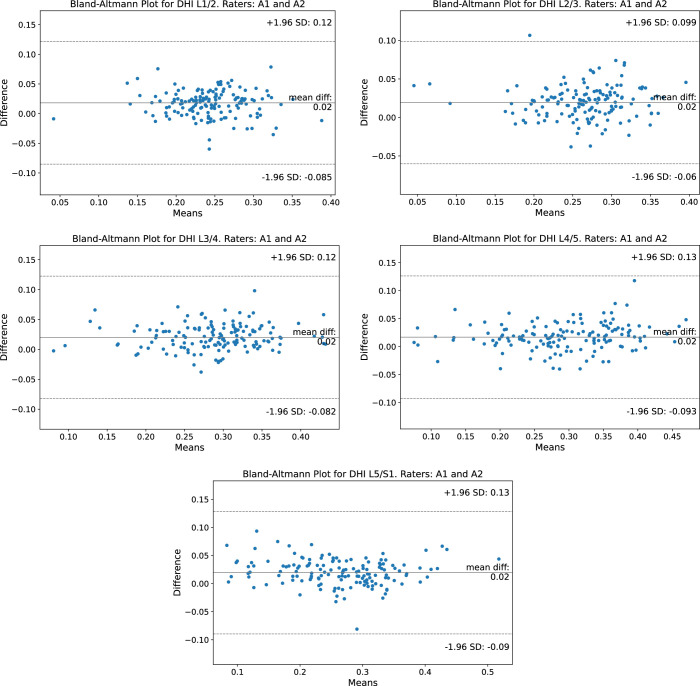
Bland-Altman plot for intervertebral Disc Height Index calculations based on annotations from raters A1 and A2 across all lumbar spine levels. The plot shows a mean difference of 0.02, indicating close agreement between the two raters.

Quantitative assessments of DHI were conducted using the Bland-Altman (BA) analysis and paired *t* test. A paired *t* test showed that differences in the DHI measurements computed from ground truth labels of A1 and A2 were statistically significant (*P* < 0.05). However, BA plots (Figure 5) revealed a consistent mean difference of 0.02 across all lumbar intervertebral discs (L1/2–L5/S1), indicating minimal systematic bias between raters. The SD remained consistent across all lumbar levels, measuring 0.12 for the upper lumbar discs (L1/2, L3/4) and 0.13 for the lower lumbar discs (L4/5, L5/S1). Coefficient of determination R^2^ values indicated good interrater agreement with low variability, especially at lower lumbar levels (Table 4).

**TABLE 4:** Coefficient of Determination R^2^ Measured on the DHI Measurements From the Segmentation Masks of Annotators A1 and A2 for Each Lumbar Level L1/2 to L5/S1

Lumbar Level	Coefficient of Determination (R^2^)	95% CI
L1/2	0.78	0.71, 0.84
L2/3	0.85	0.78, 0.90
L3/4	0.89	0.85, 0.92
L4/5	0.92	0.89, 0.94
L5/S1	0.92	0.90, 0.94

95% CI is computed based on one thousand bootstrap resampling runs.

## DISCUSSION

**Figure 6:**
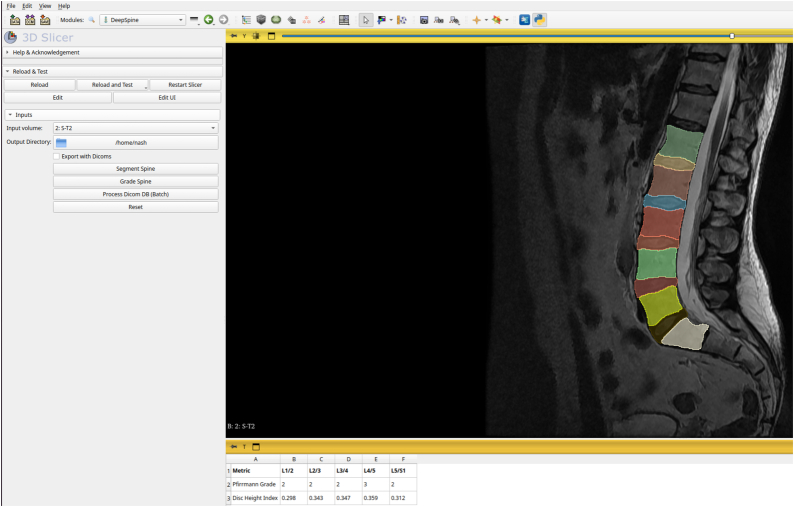
3D Slicer extension interface, showcasing the segmentation, grading, and batch processing modules. The extension enables automated analysis of lumbar spine MRI, including vertebral body and disc segmentation, grading, and Disc Height Index calculation, streamlining large-scale data processing for research and clinical applications.

This study has addressed the need for technical expertise in utilizing DL models for lumbar spine image analysis by developing an OLSIA—an interactive software platform built on top of 3D Slicer. Our software provides tools for lumbar spine segmentation, grading and DHI calculations, streamlining quantitative data analysis without requiring user scripting^22^ or DL model training.^26,27^ While OLSIA performs effectively on CPUs, processing speed is significantly enhanced by GPUs and increased RAM (Table 2). The batch processing feature is particularly beneficial for large data sets, assuming adequate GPU hardware for faster analysis. OLSIA’s user interface is shown in Figure 6.

Significant progress has been made in automated spine analysis tools. However, many existing tools focus primarily on spine segmentation,^46–48^ often using popular DL architectures like nn-Unet^49^ and TotalSegmentator^29^ or address niche tasks like dendritic spine^50^ and spinal cord^51^ segmentation. In contrast, OLSIA provides a GUI-based tool with a unified workflow for MRI-based lumbar spine segmentation, grading and DHI calculations. It employs robust deep learning models evaluated on 6 external data sets and outperforming TotalSegmentator baselines. Furthermore, OLSIA is standalone software, integratable into hospital PACS through a 3D Slicer extension, and requires no user-level model training or scripting, facilitating easier integration into research workflows.

For robust validation, we evaluated OLSIA on a compiled set of 6 diverse lumbar spine data sets (2 public, 4 private). The DL models^9^ benefited both from augmentations and model adaptation methods, showing notable DSC improvements across all data sets (Table 3), indicating more precise VB and disc detection. Scaling augmentation significantly improved performance on TwinsUK (DSC 9.5 → 86.7). Furthermore, WBN enhanced performance for TwinsUK (DSC 86.7 → 88.9) and HKDDC (DSC 88.7 → 90.5) by balancing data statistics (Table 3). Marginal gains on other data sets might suggest performance saturation, leaving limited potential for further gains.

Interrater agreement between raters A1 and A2, measured using the DSC, was high for the CETIR (95.0 ± 0.1), NCSD (94.3 ± 0.1), and Mendeley (95.4 ± 0.0) data sets. The TwinsUK (93.8 ± 0.1) and SPIDER (92.9 ± 0.5) data sets showed slightly lower DSC values. Visual inspection of the annotations revealed that this discrepancy stems from variations in sacral segmentation in cases with lumbosacral transitional vertebrae.^52^ Despite a statistically significant difference (*P* < 0.05) in the measurement of DHI between the annotations of A1 and A2, BA plots revealed a relatively small mean difference (0.02), indicating low systematic bias.

Despite these improvements, there are some limitations to OLSIA. First, our DL models^9^ are trained on T2-weighted fat-suppressed 2D mid and parasagittal images of the lumbar spine. Therefore, OLSIA is limited to the segmentation of these regions in the DICOM series. Second, NFBC1966 training data features a homogenous age group and exclusively lumbar spine scans. While the model was evaluated on 6 external data sets (cohort-based, clinical, and symptomatic), full spine, pediatric MRI and anatomic variations such as lumbosacral transitional vertebrae^52^ may require further development. Third, OLSIA currently processes only T2-weighted MRI scans, although future incorporation of other modalities could enhance its robustness and applicability.

## CONCLUSIONS

We developed OLSIA and validated it on 6 diverse external data sets. Its easy-to-use tools for automated segmentation, grading, and DHI calculation empower researchers without DL expertise. OLSIA can accelerate LSMRI analysis and radiomics research, contributing to understanding spinal disorders. Future work will focus on expanding OLSIA’s capabilities to include the measurement of facet tropism.^53^ OLSIA is available at https://imedslab.github.io/spineslicer/.

Key Points

- We developed Open Lumbar Spine Image Analysis (OLSIA)—an open platform for lumbar spine analysis featuring spine segmentation, grading, and intervertebral Disc Height Index (DHI) calculation.
- The models were trained on the North Finland Birth Cohort 1966 data set and externally validated across 6 geographically diverse data sets, showing strong generalizability.
- Bland-Altman plots reveal a low systemic bias (mean difference: 0.2) for DHI.
- Facilitates researchers across disciplines to leverage the platform’s no-code tools for lumbar spine segmentation, grading and DHI calculations.

## Supplementary Material
