# Supplementary material for: Open Lumbar Spine Image Analysis: A 3D Slicer Extension for Segmentation, Grading, and Intervertebral Disc Height Index With Multi–Data Set Validation
Source: Spine (Phila Pa 1976). 2025 Aug 1;51(2):E35–44. doi: 10.1097/BRS.0000000000005462 (PMC12695293; doi:10.1097/BRS.0000000000005462)
Supplement: Supplementary file 1 [file brs-51-e35-s001.docx]

**Base Model Architectures:**

In our previous study, we evaluated several deep learning architectures for both spine segmentation and grading. In the case of segmentation, Feature Pyramid Network based architecture with resnet-34 encoder was chosen whereas for classification of disc degeneration using Pfirrmann grades, EfficientNetB0 was chosen. In our current study, these architectures are referred to as base models.

**Supplementary Table 1:** Scan parameters T2-weighted scans of the lumbar spine across all the datasets. NFBC^1^1966 is used for training of OLSIA^2^ application and other datasets are used for evaluation purposes.

| **Dataset** | **Country** | **Manufacturer** | **Image Matrix** | **Pixel Spacing** | **Slice Thickness** |
| --- | --- | --- | --- | --- | --- |
| NFBC1966 | Finland | 1.5 Tesla GE Signa HDxt (General Electric, Milwaukee, WI, USA) | 512x512 | 0.3 mm | 4.0 mm |
| TwinsUK | United Kingdom | 1.0T (Siemens, Munich, Germany)  1.5T (Siemens, Munich, Germany) | 448x448 to 512x512 | 0.93mm/0.85mm | 4.0 mm |
| HKDDC^3^ | Hong Kong | 1.5T scanner (Siemens, Munich, Germany or Philips, Best, The Netherlands) | 448x448 to 512x512 | 0.670 mm | 4.0 mm |
| CETIR | Spain | 1.5T GE Medical Systems (Chicago, Illinois, USA) | 512 x 512 | 0.68 mm | 4.4 mm |
| NCSD | Hungary | 0.4T Hitachi Aperto (Tokyo, Japan) | 512 x 512 | 0.68 mm | 4.0 mm |
| Mendeley* | Around the world | 1.5 T Siemens Magnetom Essenza | 384 x 384 | 0.72 mm | 4.0 mm |
| SPIDER* | Netherlands | 1.5-T Siemens | 512 x 512 | 0.3mm to 0.9mm | 3.3 mm to 4.8 mm |

^1^Northern Finland Birth Cohort

^2^Open Lumbar Spine Image Analysis

^3^Hong Kong Disc Degeneration Cohort

^*^Open datasets available for download
